# Galiellalactone inhibits the STAT3/AR signaling axis and suppresses Enzalutamide-resistant Prostate Cancer

**DOI:** 10.1038/s41598-018-35612-z

**Published:** 2018-11-23

**Authors:** Daksh Thaper, Sepideh Vahid, Ramandeep Kaur, Sahil Kumar, Shaghayegh Nouruzi, Jennifer L. Bishop, Martin Johansson, Amina Zoubeidi

**Affiliations:** 1 Vancouver Prostate Centre, Vancouver, BC Canada; 2 Department of Urologic Sciences, Faculty of Medicine, University of British Columbia, Vancouver, BC Canada; 3 Glactone Pharma AB, Helsingborg, Sweden

## Abstract

Most prostate cancer patients will progress to a castration-resistant state (CRPC) after androgen ablation therapy and despite the development of new potent anti-androgens, like enzalutamide (ENZ), which prolong survival in CRPC, ENZ-resistance (ENZ^R^) rapidly occurs. Re-activation of the androgen receptor (AR) is a major mechanism of resistance. Interrogating our *in vivo* derived ENZ^R^ model, we discovered that transcription factor STAT3 not only displayed increased nuclear localization but also bound to and facilitated AR activity. We observed increased STAT3 S727 phosphorylation in ENZ^R^ cells, which has been previously reported to facilitate AR binding. Strikingly, ENZ^R^ cells were more sensitive to inhibition with STAT3 DNA-binding inhibitor galiellalactone (GPA500) compared to CRPC cells. Treatment with GPA500 suppressed AR activity and significantly reduced expression of Cyclin D1, thus reducing cell cycle progression into S phase and hindering cell proliferation. *In vivo*, GPA500 reduced tumor volume and serum PSA in ENZ^R^ xenografts. Lastly, the combination of ENZ and GPA500 was additive in the inhibition of AR activity and proliferation in LNCaP and CRPC cells, providing rationale for combination therapy. Overall, these results suggest that STAT3 inhibition is a rational therapeutic approach for ENZ^R^ prostate cancer, and could be valuable in CRPC in combination with ENZ.

## Introduction

With 1 in 8 Canadian males expected to be diagnosed with prostate cancer (PCa) in their lifetime and 23,600 new cases in 2014 in Canada alone, PCa is the most diagnosed cancer among Canadian males^1^. Despite early detection and localized surgery, cancer recurs in a significant number of patients and Androgen Deprivation Therapy (ADT) is used to block the growth-promoting effects of the Androgen Receptor (AR) and decrease tumor burden in this population. Though initially effective, the tumors eventually become castration resistant (CRPC) by replenishing AR activity^2^. Consequently, further use of AR Pathway Inhibitors (ARPI) is the cornerstone of CRPC treatment developed over the last decade. Enzalutamide (ENZ), a second generation anti-androgen, has significantly improved survival of CRPC patients^3^. However, as observed with ADT, the efficacy of ENZ treatment is short-lived and tumors become resistant^4,5^. As such, there is currently high demand for understanding the mechanisms driving ENZ resistance in these patients. Previous data from our laboratory demonstrates that, similar to the mechanisms of resistance in CRPC, ENZ resistance is often coupled with reactivation of AR. Under these conditions, AR activity can persist due to AR mutations, increase in steroidogenesis as well as altered expression and activation of co-regulators^6–8^. In this study, we attempt to investigate the role of Signal Transducer and Activator of Transcription 3 (STAT3) in ENZ resistance as a binding partner that can facilitate AR activity^9^.

STAT3 is a protein hub for several oncogenic signalling pathways and regulates the expression of key effectors in tumor cell survival (Bcl-xL, Bcl-2, Mcl-1)^10^, proliferation (Cyclin D1, D2, c-Myc)^11,12^, angiogenesis (bFGF, VEGF)^13,14^ and metastasis^15^. The canonical STAT3 pathway is identified by phosphorylation of STAT3 on Tyrosine 705 (Y705) (typically through Janus Associated Kinases (JAK’s) in response to cytokines from IL-6 and IL-10 family) and subsequent dimerization and nuclear localization of STAT3. Additionally, direct activation can also take place via phosphorylation by receptor tyrosine kinases (i.e. EGFR, VEGFR, IGFR) and non-receptor tyrosine kinases such as Src family kinases^16^. STAT3 activity is significant in PCa progression whether it’s treatment naïve, castrate-resistant or metastatic^17–20^. This can be attributed to STAT3’s role in regulating several driving forces of PCa progression including integration of signaling pathways involved in re-activation of AR (i.e. the PTEN/PI3k/AKT pathway)^21,22^. Furthermore, the activity of mTOR and MAPK pathways phosphorylate STAT3 at Serine 727 (S727) which results in direct interaction with the N-terminal domain of AR and enhances AR transcriptional activity^23,24^, while S727A mutation significantly reduces this interaction; hindering AR transcriptional activity and highlighting the significance of AR/STAT3 co-operation in PCa progression^25^.

Utilizing a unique model of ENZ resistance, our lab explores mechanisms of resistance that arise under the pressure of ENZ^8,26^. Studying STAT3 activity in these LNCaP-derived ENZ resistant cells, we discovered that inhibition of STAT3 by the DNA binding inhibitor galiellalactone (GPA500)^27,28^ disrupted the interaction between STAT3 and AR, reduced AR activity and reduced cell proliferation in PSA producing ENZ-resistant cells (PSA^hi^ ENZ^R^). Furthermore, combination of ENZ and GPA500 in LNCaP and CRPC cells *in vitro* had an additive effect on AR inhibition and cell proliferation. These effects translated well *in vivo;* GPA500 treatment reduced tumor volume of ENZ^R^ xenografts and reduced serum PSA levels. Taken together, our study provides proof-of-principle that STAT3 inhibition using galiellalactone is a viable treatment option as monotherapy in ENZ^R^ prostate cancer as well as a logical strategy for combination therapy with ENZ in CRPC.

## Results

### STAT3 is nuclear and is co-localized with AR in PSA^hi^ ENZ^R^ cells

**Figure 1:**
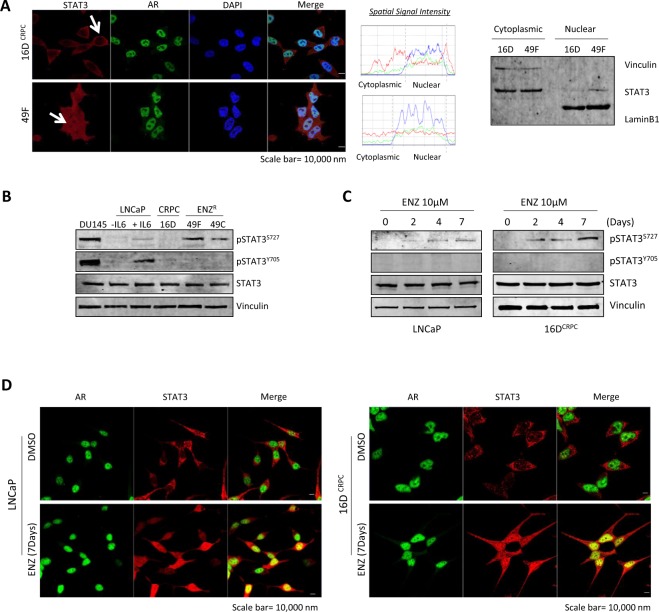
STAT3 is nuclear and is co-localized with AR in PSA^hi^ ENZ^R^ cells. (**A**) (Left) Immunofluorescence of STAT3 (red), AR (green) and the nucleus (DAPI, blue) in 16D CRPC and ENZ^R^ 49 F cells. Scale bar: 10 μm. (Middle) Graph visualization of nuclear and cytoplasmic levels of STAT3 and AR by calculating Spatial Signal Intensity. (Right) Cytoplasmic/Nuclear fractionation of STAT3, Vinculin and LaminB1 in 16D and 49 F cells. (**B**,**C**) Protein expression of p-STAT3^S727^, p-STAT3^Y705^, STAT3 and Vinculin in representative cell lines (**B**) DU145, LNCaP cells treated with/without IL6 (50 ng/mL), 16D CRPC, ENZ^R^ 49 F and 49 C and (**C**) LNCaP and 16D CRPC cells treated with 10 μM ENZ for indicated days. (**D**) Immunofluorescence of AR (green) and STAT3 (red) in LNCaP (Left) and 16D CRPC (Right) cells treated with 10 µM ENZ for 7 days. Scale bar: 10 μm.

As with most transcription factors, STAT3’s primary activity occurs upon translocation into the nucleus. Interestingly, comparison of STAT3 localization in 16D^CRPC^ and PSA^hi^ ENZ^R^ cells (49 C and 49 F) showed that not only is STAT3 more localized to the nucleus in ENZ^R^ cells compared to CRPC, but also there is a clear co-localization between STAT3 and AR in these cells (Fig. 1A left, Supplementary Fig. S1). Nuclear localization of STAT3 in ENZ^R^ cells was further confirmed using a cytoplasmic/nuclear fractionation (Fig. 1A right). Conventionally, Y705 phosphorylation was considered as the prequel to S727 phosphorylation and STAT3 activation^29^; therefore, we explored the phosphorylation status of STAT3 in ENZ^R^ cells. Surprisingly, in comparison to 16D^CRPC^, 49 C and 49 F cells exhibit an increase in pSTAT3 S727, but not Y705 (Fig. 1B) (DU145 and IL6-treated LNCaPs were used as positive controls). Furthermore, long-term exposure of LNCaP and 16D^CRPC^ cells to 10 µM ENZ clearly confirmed an increase in S727 phosphorylation in a time-dependent manner with no change in tyrosine phosphorylation (Fig. 1C). Strikingly, we observed a drastic increase in nuclear STAT3 and a clear co-localization with AR in LNCaP and 16D^CRPC^ cells treated with ENZ (10 µM for 7 days) (Fig. 1D). This data supports previous studies documenting that S727 phosphorylation can activate STAT3 signaling independent of Y705 phosphorylation^30,31^.

Interestingly, while ENZ^R^ cells present with more nuclear STAT3 (Fig. 1A), analysis of RNA-seq in these cell lines revealed that only a subset of canonical STAT3 pathway genes^32^ were enriched (Supplementary Fig. S2, Table S1). Instead, several non-canonical STAT3 targets were upregulated (Supplementary Fig. S3, Table S2). These findings, support data showing that STAT3 not only binds DNA without Y705 phosphorylation^33–35^, but also results in activation of non-canonical STAT3 target genes. Taken together, these results indicate ENZ resistance increases STAT3 S727 phosphorylation and activates STAT3 signaling, suggesting that these cells might employ persistent STAT3/AR signaling activity as a mechanism of resistance.

### PSA^hi^ ENZ^R^ cells are sensitive to STAT3 inhibition by gallielalactone

**Figure 2:**
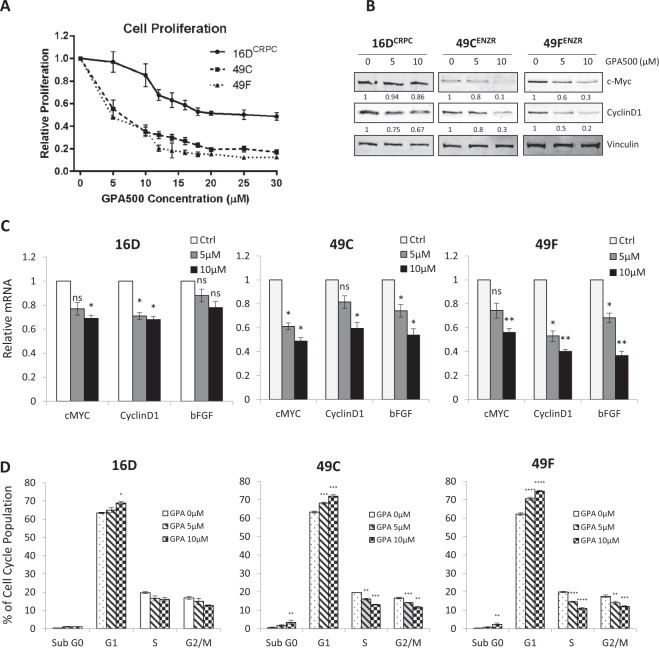
PSA^hi^ ENZ^R^ cells are sensitive to STAT3 inhibition by gallielalactone (GPA500). (**A**) Relative 72 hour cell proliferation assay using WST8 in 16D CRPC, 49 C and 49 F cells treated with indicated concentrations of GPA500 compared to untreated. (**B**) Protein expression of c-Myc, CyclinD1 and Vinculin and (**C**) Relative mRNA expression of c-Myc, CyclinD1 and bFGF in 16D^CRPC^ and PSA^hi^ ENZ^R^ cells (49 C and 49 F) treated with 0, 5 or 10 µM GPA500. (**D**) Cell cycle fraction of 16D^CRPC^ and PSA^hi^ ENZ^R^ cells 49 C and 49 F following GPA500 treatment (48 hour with indicated concentrations). All graphs represent pooled data from 3 independent experiments in triplicates.

Using our model of ENZ^R^^8,26^, we examined the response of CRPC (16D^CRPC^) and PSA^hi^ ENZ^R^ (49 C and 49 F) cells to the STAT3 inhibitor GPA500 and found that the PSA^hi^ ENZ^R^ cells were more sensitive to STAT3 inhibition in comparison to 16D^CRPC^ (Fig. 2A). Interrogating this effect, we discovered that GPA500 reduces the expression of canonical STAT3 target genes, Cyclin D1 and C-Myc, at the protein (Fig. 2B) and mRNA levels (Fig. 2C) more potently in ENZ^R^ cells. Moreover, we observed significant reduction in the mRNA levels of basic fibroblast growth factor (bFGF), a STAT3-regulated growth factor that promotes cell proliferation and angiogenesis (Fig. 2C).

Given the crucial role of Cyclin D1 in cell cycle progression, reduction of Cyclin D1 induces a G1 phase arrest^11^. In harmony with these findings, we discovered that GPA500 treatment in ENZ^R^ cells 49 C and 49 F triggered a more significant G1 phase arrest and a subsequent reduction of cells in S and G_2_/M phases in comparison to 16D^CRPC^ cells (Fig. 2D). This reduction was also accompanied by a small increase in the sub-G_0_ fraction (Fig. 2D). Overall, these findings suggest that PSA^hi^ ENZ^R^ cells, 49 C and 49 F, are dependent on STAT3 as an important proliferative transcription factor and are more sensitive to STAT3 inhibition when compared to CRPC cells.

### Inhibition of STAT3 reduces AR activity in PSA^hi^ ENZ^R^ cells *in vitro*

**Figure 3:**
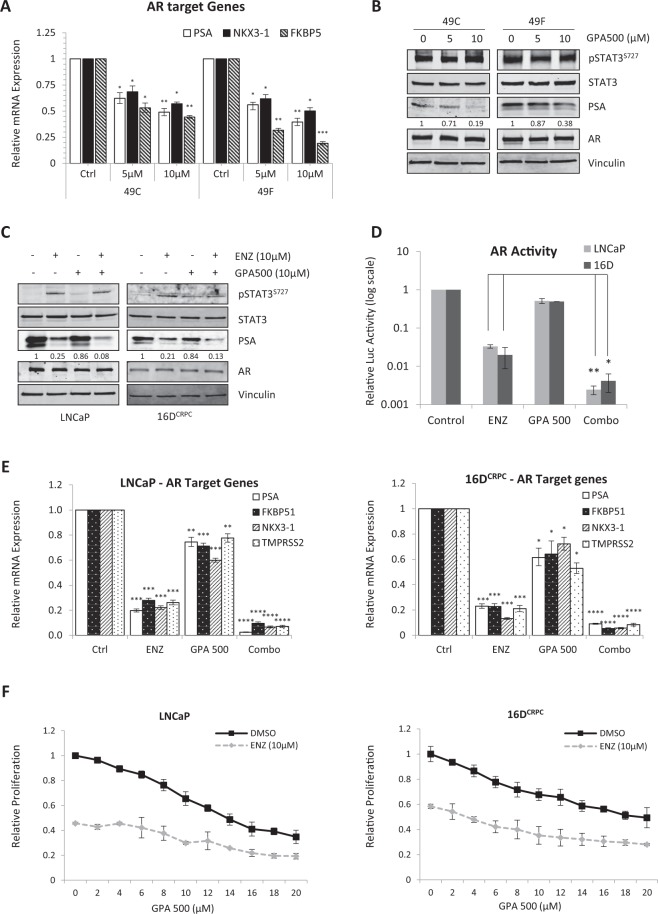
Inhibition of STAT3 reduces AR activity in PSA^hi^ ENZ^R^ cells. (**A**) Relative mRNA expression of AR target genes PSA, NKX3-1 and FKBP5 and (**B**) Protein expression of p-STAT3^S727^, STAT3, PSA, AR and Vinculin in PSA^hi^ ENZ^R^ cells 49 C and 49 F treated with 0, 5 or 10 µM GPA500 for 48 hours. (**C**) Protein expression of p-STAT3^S727^, STAT3, PSA, AR and Vinculin and (**D**) Relative AR activity assessed by luciferase assay and (**E**) Relative mRNA expression of AR target genes PSA, FKBP5, NKX3-1 and TMPRSS2 in LNCaP (Left) and 16D CRPC cells (Right) treated with 10 µM ENZ or GPA500 or combination of both for 48 hours. (**F**) Relative 72 hour cell proliferation assay using WST8 in LNCaP (Left) and 16D CRPC cells (Right) treated with indicated ENZ and GPA500 concentration. All graphs represent pooled data from 3 independent experiments and relative luciferase activity is compared to non-treated samples (Control = 1).

Re-activation of AR is one of the major factors in the emergence of ENZ resistant prostate cancer. In our model of ENZ^R^^8^, we discovered that 75% of resistant tumors regained AR activity. To further explore the relationship between AR and STAT3 in these cell lines, we tested for changes in AR activity upon STAT3 inhibition. GPA500 treatment decreases the expression of AR target genes in PSA^hi^ ENZ^R^ cells, 49 C and 49 F, in a dose dependent manner (Fig. 3A) and reduced AR activity (Supplementary Fig. S4A) without altering S727 phosphorylation (Fig. 3B). More interestingly, we found that there’s less interaction between STAT3 and AR in the presence of GPA500 (Supplementary Fig. S4B).

To investigate the effects of simultaneous AR/STAT3 inhibition, LNCaP and 16D^CRPC^ cells were treated with GPA500 or ENZ alone and in combination. Consistent with our previous results (Fig. 2C), we found that ENZ treatment induced Ser727 phosphorylation of STAT3 and decreased PSA in LNCaP and 16D^CRPC^ cells without affecting AR expression (Fig. 3C). Also, combination of both GPA500 and ENZ further reduced PSA (Fig. 3C). This data was supplemented by an AR transactivation assay (Fig. 3D) and qRT-PCR of AR target genes PSA, FKBP51, NKX3-1, TMPRSS2 (Fig. 3E). Combination of both drugs reduced AR activity more than either monotherapy in LNCaP (Fig. 3E Left) and 16D^CRPC^ cells (Fig. 3E Right). Finally, in a cell growth assay for these cell lines we discovered GPA500 and ENZ reduced cell proliferation in an additive manner (Fig. 3F). These results suggest that STAT3 may play an important role for growth of PCa cells under pressure of anti-androgen treatment and co-targeting both pathways may provide additional effect.

### Inhibition of STAT3 reduces AR activity in PSA^hi^ ENZ^R^ cells *in vivo*

**Figure 4:**
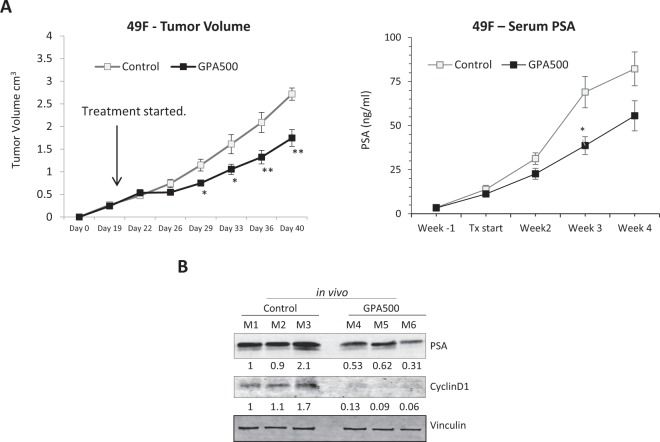
Inhibition of STAT3 reduces AR activity in PSA^hi^ ENZ^R^ cells *in vivo*. (**A**) Weekly mean tumour volume (Left) and serum prostate-specific antigen (PSA) levels (Right) in mice treated with GPA500 5 mg/kg/day versus vehicle. Treatment was started when tumours reached 200 mm^3^ and was given intraperitoneally for 5 days on and 2 days off. (**B**) Protein expression of PSA, CyclinD1 and Vinculin in collected tumors treated with vehicle (control M1, M2 and M3) or GPA500 5 mg/kg (M4, M5 and M6).

The efficacy of GPA500 on PSA^hi^ ENZ^R^ 49 F cells that were subcutaneously injected into castrated male nude mice was tested. Once the tumor volume reached 200 mm^3^, mice were randomized and treated with vehicle control or 5 mg/kg/day of GPA500. Consistent with our *in vitro* data (Fig. 2A), treatment with GPA500 reduced tumor growth compared to control (Fig. 4A). Consequently, the serum PSA in the treated mice was also lower (Fig. 4B). The reduction in serum PSA could be attributed to the lower tumor volume; as such we tested the expression of PSA in the tumors. PSA expression in the tumors of mice treated with GPA500 was lower than control (Fig. 4C). Additionally, the expression of Cyclin D1 was also reduced in the tumors treated with GPA500 (Fig. 4C) which recapitulated our previous findings *in vitro* (Fig. 2C Right). In summary, our findings suggest that targeting STAT3 in PSA producing ENZ-resistant tumors is a rational approach to reduce tumor burden.

## Discussion

Prostate cancer is the 2^nd^ most diagnosed cancer in men around the world and unfortunately, development of treatment resistance is the inevitable fate of prostate cancer patients put on 2^nd^ generation ARPI’s like ENZ. Exploring and understanding the various mechanisms of resistance that contribute to AR re-activation after receiving ENZ may guide us in designing rational treatment regimens in the future. To this end, this study shows that in our model of ENZ resistance the STAT3/AR interaction facilitates not only sustained AR activity but also STAT3 signaling. Moreover, inhibition with the STAT3 DNA-binding inhibitor galiellalactone (GPA500)^28^ suppresses both AR and STAT3 target genes. We also demonstrate that despite the conventional dogma that STAT3 dimerization and nuclear translocation requires Y705 phosphorylation, the STAT3/AR complex in ENZ-resistant cell lines can occur independent of phospho-Y705. Ultimately, we demonstrate that GPA500 shows efficacy in ENZ-resistant tumors as monotherapy *in vivo* and it displays potential for combination with Enzalutamide at earlier stages of the disease *in vitro*.

Over the past 2 decades, STAT3 activity has been repeatedly implicated in PCa initiation and progression. Loss of the tumor suppressor PTEN is the most frequent deletion in PCa and combining PTEN loss with STAT3 activation accelerates development of adenocarcinoma^36^. STAT3 is intricately linked to key transcription factors associated with PCa development such as HIF1α^37^ and NF-κB^38^ resulting in regulation of multiple oncogenic proteins including but not limited to HER2, BCL-2 and BCL-3, VEGF and TWIST1^39^. Moreover, there is a considerable body of work evaluating the interplay between STAT3 and AR, the fundamental driver of PCa. For example, IL-6 treatment and subsequent signaling through the canonical JAK/STAT3 pathway can induce AR expression/activity^21^. STAT3 was also shown to enhance AR signaling through direct interaction with the N-terminus domain of the AR^23^. Moreover, the crucial mediator in this direct interaction was found to be S727 phosphorylation on STAT3^25^ which was shown to be a driver of tumorigenesis in prostate cancer independent of Y705 phosphorylation^30^. Interestingly, S727 phosphorylation was found to be expressed more in higher Gleason Score tissues^17,25,30^ further establishing its clinical importance in localized PCa.

STAT3 signaling also plays a role in treatment-resistant prostate cancer phenotypes such as CRPC^40^, regulation of cancer stem cell phenotype^41^ as well as development of resistance to 2^nd^ generation anti-androgen, ENZ^42^. An investigation by Liu *et al*. showed that constitutive activation of STAT3 can overcome the anti-proliferative effects of ENZ and STAT3 inhibition in LNCaP cells reverses ENZ-resistance^43^. While insightful, this data doesn’t reflect the natural progression to ENZ resistance. The effect on STAT3 was not evaluated as a response to ENZ, rather, the authors studied the reduced efficacy of ENZ in response to artificially activated STAT3^43^.

Studying the cell lines from our naturally derived ENZ-resistance tumors^8^ we found that similar to castration resistance^16^, the STAT3 pathway still plays a considerable role in ENZ resistance. Similar to LNCaP cells which they are originated from, there is little to no canonical IL6/STAT3 activity in these PSA^hi^ ENZ^R^ cells; however, pSTAT3 S727 and not Y705 is upregulated compared to 16D^CRPC^ cells. Previous research from our lab demonstrated that two of the kinase pathways upstream of Ser727 phosphorylation, ERK (MAPK1/2) and AKT, are activated in response to ENZ treatment^6^, possibly explaining the upregulation of this phosphorylation in response to ENZ. Interestingly, another group showed that treatment of LNCaP cells with ENZ does not affect the tyrosine phosphorylation of STAT3^44^, although they didn’t examine the S727 phosphorylation.

Resistance acquired against ENZ is a collection of complex events comprised of both AR dependent (direct or indirect) and AR independent mechanisms^45^. Supplementing the previously identified AR/STAT3 interaction, our study reveals that in PSA^hi^ ENZ^R^ cells, STAT3 binds AR and leads to continued expression of both AR and STAT3 target genes. However, it is important to note that this association does not upregulate all canonical STAT3 genes. Evidence for this is apparent in the RNA-seq data where only a subset of classic STAT3 genes are upregulated in the ENZ^R^ cells and the canonical pathway is not enriched overall. The increased sensitivity of the ENZ^R^ cells to STAT3 inhibition may be due to the concomitant reduction of both STAT3 and AR target genes. Indeed, combination of ENZ and galiellalactone on LNCaP and CRPC derived cells had an additive effect on AR inhibition and reduced cell proliferation more than ENZ monotherapy. In summary, our study further establishes STAT3’s pivotal role in development of ENZ resistance as it can promote oncogenic signaling not only through its canonical pathway, but also by re-activation of AR and shows that STAT3 inhibitor, galiellalactone, decreases ENZ-resistant cell proliferation *in vitro* and *in vivo*.

## Materials and Methods

### Generation of ENZ^R^ Xenografts and Cell Lines

The detailed procedure for generation of CRPC and ENZ^R^ tumors and cell lines can be found in our previously published report^8,26^.

### Cell Line Culture and Reagents

LNCaP cells were kindly provided by Dr. Leland W.K. Chung (Emory University) and authenticated in January 2013. CRPC and ENZR cell lines were generated from LNCaP cells, tested, and authenticated by whole-genome and whole-transcriptome sequencing (Illumina Genome Analyzer IIx, 2012). Cells were maintained in RPMI-1640 (+10 μmol/L ENZ (Haoyuan Chemexpress) for ENZ^R^ or DMSO (Sigma-Aldrich) for LNCaP and 16D^CRPC^. Cells were seeded at a density of 1 × 10^6^ cells/10 mL media, treated the following day with galiellalactone (GPA500, Glactone Pharma) and harvested after 48 hours unless otherwise noted.

### Cell Growth Assay

16D^CRPC^ (without ENZ in media), 49C ENZ^R^ and 49F ENZ^R^ (with ENZ in media) cells were plated in 96 well plates (Corning) at 4000 cells per well. Treatments were done next day, proliferation was quantified using WST-8 assay (Dojindo) as per manufacturer’s protocol.

### RNA Sequencing

RNA sequencing data was extracted from microarray gene expression previously performed^8^.

### Quantitative reverse transcription PCR (qRT-PCR)

Total RNA was extracted from cells using TRIzol reagent (Life Technology) and 2 μg was reversed-transcribed using MMLV reverse transcriptase and random hexamers (Invitrogen). Real-time PCR was performed using SyberGreen ROX Master Mix (Roche Applied Science). Target gene expression was normalized to GAPDH levels in three experimental replicates per sample. For primer sequences, please see Supplementary Table (Table S3).

### Luciferase Assay

Luciferase reporter assay was performed as described previously^46^. In short, 50,000 cells per well are plated in 12-well plates are transfected with ARR3-luc reporter using Mirus TransIT 20/20. 24 hours later, cells are treated and lysed for bioluminescence analysis 48 hours later.

### Cell cycle analysis using Flow Cytometry

49C^ENZR^ and 49F^ENZR^ (with ENZ) cells were plated in 10 cm dishes and treated with ENZ and/or GPA500 at indicated concentration. After incubation for 48 hr, cell-cycle fraction was analyzed using Propidium Iodide (PI). Briefly, cells were trypsinized, washed with cold PBS and fixed with 70% ethanol while light vortexing to prevent clumping. Cells were then stained with PI staining solution containing RNase. Stained cells were run through FACS CANTO II machine and analyzed using FlowJo.

### Immunoprecipitation and Western Blotting

Whole-cell extracts were obtained upon lysis of cells in cold RIPA buffer (Thermo) with 1x concentration of PhoSTOP and Protease Inhibitor (Roche). Once protein concentration was determined using BCA assay (Thermo), 40 μg samples were boiled for 5 mins in SDS sample buffer and ran on SDS-PAGE gel. Immunoprecipitation was performed using ImmunoCruz™ IP/WB Optima B System (Santa Cruz) based on the manufacturer’s guideline. 2 µg of primary antibody, or immunoglobulin G (IgG) was used for immunoprecipitation and control respectively. Transfer was done onto PVDF membranes, blocked with Odyssey Blocking Buffer (Licor), probed with primary antibodies at 1:1000 dilution. Antibodies for STAT3, pSTAT3 S727 and Y705, PSA, c-MYC, Cyclin D1 and PARP were all obtained from Cell Signaling. Vinculin antibody was purchased from Sigma while AR and LaminB1 were purchased from Santa Cruz. Image acquisition and intensity quantifications were carried out using Licor Odyssey Scanner with Application Software V3.0 and normalized to Vinculin intensity. Full length blots are provided as Supplementary Figs S5–S9.

### Immunofluorescence

Cells were plated at 25,000 per in 12 well plates on circular glass slides in a 12 well plate. Cells were fixed and stained as previously described^47^ using antibodies against STAT3 (1:100) and AR (1:100). DAPI was used to visualize nuclei and then the pictures were taken using Zeiss LSM confocal microscope. Results are representative of random pictures taken from three independent experiments.

### Animal treatment

All experimental protocols used in this study were approved by the Canadian Council on Animal Care and the University of British Columbia (UBC) Animal Care Committee (certificate number A106-0246). All methods carried out in this study are in accordance with guidelines and regulations of both organizations. Castrated male athymic mice (Sprague Dawley; Harlan, Inc., Indianapolis, IN) were injected subcutaneously with 1 × 10^6^ 49F^ENZR^ cells (suspended in 0.1 mL Matrigel; BD Biosciences). Once tumors were 200 mm^3^, mice were randomly assigned to (i) vehicle, (ii) GPA500 (5 mg/kg) and treated Intraperitoneally 5 times per week. Tumor volume and PSA measurements were performed once weekly. Serum PSA measurements were performed by cobas e 411 analyzer (Roche). After sacrifice, tumors were harvested.

### Statistical Analysis and Data Representation

In bar graphs, unpaired, two-tailed, Student t tests were performed to analyze statistical significance between groups using GraphPad Prism (GraphPad Software). Significance is indicated as follows: *P < 0.05; **P < 0.01; ***P < 0.001; ****P < 0.0001. Graphs show pooled data with error bars representing SEM obtained from at least three independent experiments.

## Electronic supplementary material

Supplementary Data

## References

[1] CCSsACoC, S. Canadian cancer statistics 2014. *Toronto*, *ON: Canadian Cancer Society* **2014** (2014).

[2] Hotte SJ, Saad F (2010). Current management of castrate-resistant prostate cancer. Curr Oncol.

[3] Scher HI (2012). Increased survival with enzalutamide in prostate cancer after chemotherapy. The New England journal of medicine.

[4] Watson PA, Arora VK, Sawyers CL (2015). Emerging mechanisms of resistance to androgen receptor inhibitors in prostate cancer. Nat Rev Cancer.

[5] Antonarakis ES (2014). AR-V7 and resistance to enzalutamide and abiraterone in prostate cancer. N Engl J Med.

[6] Toren P, Kim S, Johnson F, Zoubeidi A (2016). Combined AKT and MEK Pathway Blockade in Pre-Clinical Models of Enzalutamide-Resistant Prostate Cancer. PLoS One.

[7] Toren P (2015). Combination AZD5363 with Enzalutamide Significantly Delays Enzalutamide-resistant Prostate Cancer in Preclinical Models. Eur Urol.

[8] Bishop JL (2017). The Master Neural Transcription Factor BRN2 Is an Androgen Receptor-Suppressed Driver of Neuroendocrine Differentiation in Prostate Cancer. Cancer Discov.

[9] Yamamoto T (2003). Molecular interactions between STAT3 and protein inhibitor of activated STAT3, and androgen receptor. Biochemical and biophysical research communications.

[10] Fukada T (1996). Two signals are necessary for cell proliferation induced by a cytokine receptorgp130: involvement of STAT3 in anti-apoptosis. Immunity.

[11] Leslie K (2006). Cyclin D1 is transcriptionally regulated by and required for transformation by activated signal transducer and activator of transcription 3. Cancer Res.

[12] Bromberg JF (1999). Stat3 as an oncogene. Cell.

[13] Niu G (2002). Constitutive Stat3 activity up-regulates VEGF expression and tumor angiogenesis. Oncogene.

[14] Huang YH (2013). STAT1 activation by venous malformations mutant Tie2-R849W antagonizes VEGF-A-mediated angiogenic response partly via reduced bFGF production. Angiogenesis.

[15] Huang S (2007). Regulation of metastases by signal transducer and activator of transcription 3 signaling pathway: clinical implications. Clin Cancer Res.

[16] Bishop JL, Thaper D, Zoubeidi A (2014). The Multifaceted Roles of STAT3 Signaling in the Progression of Prostate Cancer. Cancers.

[17] Cocchiola R (2017). Analysis of STAT3 post-translational modifications (PTMs) in human prostate cancer with different Gleason Score. Oncotarget.

[18] Don-Doncow N (2017). Expression of STAT3 in Prostate Cancer Metastases. Eur Urol.

[19] Tam L (2007). Expression levels of the JAK/STAT pathway in the transition from hormone-sensitive to hormone-refractory prostate cancer. Br J Cancer.

[20] Mohanty SK (2017). STAT3 and STAT5A are potential therapeutic targets in castration-resistant prostate cancer. Oncotarget.

[21] Yang L (2003). Interleukin-6 differentially regulates androgen receptor transactivation via PI3K-Akt, STAT3, and MAPK, three distinct signal pathways in prostate cancer cells. Biochemical and biophysical research communications.

[22] Lin DL, Whitney MC, Yao Z, Keller ET (2001). Interleukin-6 induces androgen responsiveness in prostate cancer cells through up-regulation of androgen receptor expression. Clinical cancer research: an official journal of the American Association for Cancer Research.

[23] Ueda T, Bruchovsky N, Sadar MD (2002). Activation of the androgen receptor N-terminal domain by interleukin-6 via MAPK and STAT3 signal transduction pathways. The Journal of biological chemistry.

[24] Chen T, Wang LH, Farrar WL (2000). Interleukin 6 activates androgen receptor-mediated gene expression through a signal transducer and activator of transcription 3-dependent pathway in LNCaP prostate cancer cells. Cancer Res.

[25] Hsu FN (2013). Cyclin-dependent kinase 5 modulates STAT3 and androgen receptor activation through phosphorylation of Ser(7)(2)(7) on STAT3 in prostate cancer cells. Am J Physiol Endocrinol Metab.

[26] Kuruma H (2013). A novel antiandrogen, Compound 30, suppresses castration-resistant and MDV3100-resistant prostate cancer growth *in vitro* and *in vivo*. Mol Cancer Ther.

[27] Hellsten R (2008). Galiellalactone is a novel therapeutic candidate against hormone-refractory prostate cancer expressing activated Stat3. Prostate.

[28] Don-Doncow N (2014). Galiellalactone is a direct inhibitor of the transcription factor STAT3 in prostate cancer cells. J Biol Chem.

[29] Yu H, Lee H, Herrmann A, Buettner R, Jove R (2014). Revisiting STAT3 signalling in cancer: new and unexpected biological functions. Nat Rev Cancer.

[30] Qin HR (2008). Activation of signal transducer and activator of transcription 3 through a phosphomimetic serine 727 promotes prostate tumorigenesis independent of tyrosine 705 phosphorylation. Cancer Res.

[31] Androutsellis-Theotokis A (2006). Notch signalling regulates stem cell numbers *in vitro* and *in vivo*. Nature.

[32] Azare J (2007). Constitutively activated Stat3 induces tumorigenesis and enhances cell motility of prostate epithelial cells through integrin beta 6. Molecular and cellular biology.

[33] Yang J (2005). Novel roles of unphosphorylated STAT3 in oncogenesis and transcriptional regulation. Cancer Res.

[34] Yang J, Stark GR (2008). Roles of unphosphorylated STATs in signaling. Cell research.

[35] Cimica V, Chen HC, Iyer JK, Reich NC (2011). Dynamics of the STAT3 transcription factor: nuclear import dependent on Ran and importin-beta1. PloS one.

[36] Smith DA, Kiba A, Zong Y, Witte ON (2013). Interleukin-6 and oncostatin-M synergize with the PI3K/AKT pathway to promote aggressive prostate malignancy in mouse and human tissues. Molecular cancer research: MCR.

[37] Pawlus MR, Wang L, Hu CJ (2014). STAT3 and HIF1alpha cooperatively activate HIF1 target genes in MDA-MB-231 and RCC4 cells. Oncogene.

[38] Yu Z, Zhang W, Kone BC (2002). Signal transducers and activators of transcription 3 (STAT3) inhibits transcription of the inducible nitric oxide synthase gene by interacting with nuclear factor kappaB. The Biochemical journal.

[39] Carpenter RL, Lo HW (2014). STAT3 Target Genes Relevant to Human Cancers. Cancers (Basel).

[40] Shiota M (2013). Hsp27 regulates epithelial mesenchymal transition, metastasis, and circulating tumor cells in prostate cancer. Cancer Res.

[41] Schroeder A (2014). Loss of androgen receptor expression promotes a stem-like cell phenotype in prostate cancer through STAT3 signaling. Cancer Res.

[42] Wang C (2018). Blocking the Feedback Loop between Neuroendocrine Differentiation and Macrophages Improves the Therapeutic Effects of Enzalutamide (MDV3100) on Prostate Cancer. Clin Cancer Res.

[43] Liu C (2014). Inhibition of constitutively active Stat3 reverses enzalutamide resistance in LNCaP derivative prostate cancer cells. Prostate.

[44] Handle F (2016). SOCS3 Modulates the Response to Enzalutamide and Is Regulated by Androgen Receptor Signaling and CpG Methylation in Prostate Cancer Cells. Mol Cancer Res.

[45] Claessens F (2014). Emerging mechanisms of enzalutamide resistance in prostate cancer. Nature reviews. Urology.

[46] Vahid S, Thaper D, Gibson KF, Bishop JL, Zoubeidi A (2016). Molecular chaperone Hsp27 regulates the Hippo tumor suppressor pathway in cancer. Scientific reports.

[47] Thaper D, Vahid S, Nip K M, Moskalev I, Shan X, Frees S, Roberts M E, Ketola K, Harder K W, Gregory-Evans C, Bishop J L, Zoubeidi A (2017). Targeting Lyn regulates Snail family shuttling and inhibits metastasis. Oncogene.

