# Supplementary material for: Galiellalactone inhibits the STAT3/AR signaling axis and suppresses Enzalutamide-resistant Prostate Cancer
Source: Sci Rep. 2018 Nov 23;8:17307. doi: 10.1038/s41598-018-35612-z (PMC6251893; doi:10.1038/s41598-018-35612-z)
Supplement: Supplementary file 1 — Supplementary Data [file 41598_2018_35612_MOESM1_ESM.pdf]

Galiellalactone inhibits the STAT3/AR signaling axis and suppresses Enzalutamide-resistant Prostate Cancer.

Daksh Thaper[1,2*], Sepideh Vahid[1,2*], Ramandeep Kaur[1], Sahil Kumar[1], Shaghayegh Nouruzi[1,2], Jennifer L. Bishop[1], Martin Johansson[3] and Amina Zoubeidi[1,2]

* Authors contributed equally.

1: Vancouver Prostate Centre, Vancouver, BC, CA.

2: University of British Columbia, Faculty of Medicine, Department of Urologic Science, Vancouver, BC, CA.

3: Glactone Pharma AB, Helsingborg, Sweden

RUNNING TITLE: Galiellalactone suppresses ENZ-resistant Prostate Cancer.

KEY WORDS: STAT3, Androgen Receptor, Enzalutamide Resistance, Prostate Cancer, Galiellalactone

CORRESPONDING AUTHOR INFORMATION:

Amina Zoubeidi, PhD (azoubeidi@prostatecentre.com)

Associate Professor, University of British Columbia Department of Urology

Vancouver Prostate Centre

2660 Oak Street

Vancouver BC V6H3Z6

Phone: (604) 875-4111 # 68880

Fax: (604) 875-5654

SUPPLEMENTARY INFO LEGENDS

Supplementary Figure S1. Co-localization of AR/STAT3 in PSA^{hi} ENZ^{R} vs. 16D^{CRPC} cells.

Immunofluorescence images of STAT3 (red), AR (green), nucleus (DAPI, blue) and merged channel in ENZ-Resistant 49C cells. Scale bar: 10 µm. Graph visualization of nuclear and cytoplasmic levels of STAT3 and AR by calculating Spatial Signal Intensity is also provided.

Supplementary Figure S2. Canonical STAT3 Upregulated Gene Signature in PSA^{hi} ENZ^{R} cells.

Gene Set Enrichment Analysis (GSEA) of canonical STAT3 target genes (upregulated) in ENZ-resistant 49C and 49F cells based on previously published STAT3 signature [32]. Enrichment scores are provided. Genes used in this analysis are listed in Supplementary Table (Table S1).

Supplementary Figure S3. Non-Canonical STAT3 Gene Signature in PSA^{hi} ENZ^{R} compared to CRPC cells.

Heat map showing non-canonical STAT3 target genes in ENZ^{R} 49F cells. Compared to 16D^{CRPC} . Expression values are listed in Supplementary Table 2 as Reads Per Kilo Million (RPKM).

Supplementary Figure S4. Galiellalactone reduces AR activity and STAT3/AR interaction in PSA^{hi} ENZ^{R} cells.

A) Relative AR luciferase activity after treatment with GPA500 at indicated doses was normalized and compared to non-treated samples. **B)** Protein expression of AR and STAT3 after immunoprecipitation of AR in ENR^{R} 49F cells treated +/- GPA500 10µM.

Supplementary Figure S5-S9.

Full length blots for Figure 1 to Figure 4 that are provided in the manuscript.

Supplementary Table S1-S3.

Supplementary Table S1: STAT3 canonical gene signature from Azare et. al [32]

Supplementary Table S2: STAT3 non-canonical gene expression in 49F ENZ resistant cells compared to 16D CRPC.

Supplementary Table S3: List of SYBR Green Primer Sequences for qRT-PCR

Supplementary Figure S1

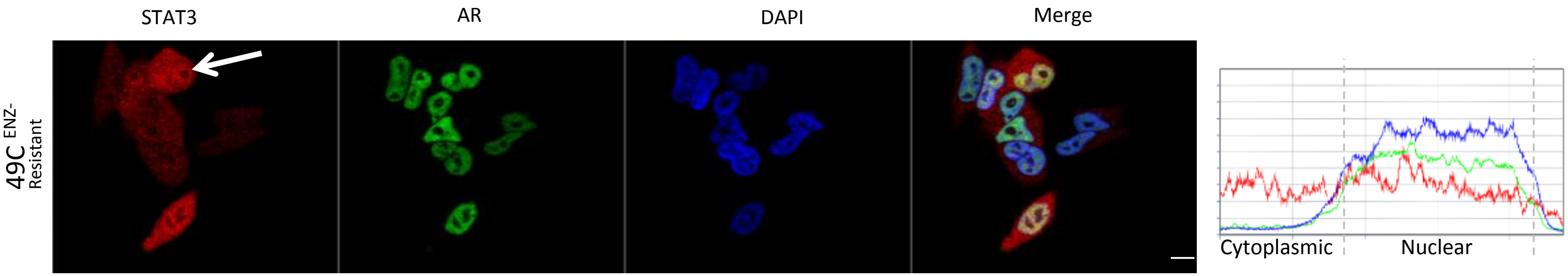

Scale bar= 10,000 nm

Supplementary Figure S2

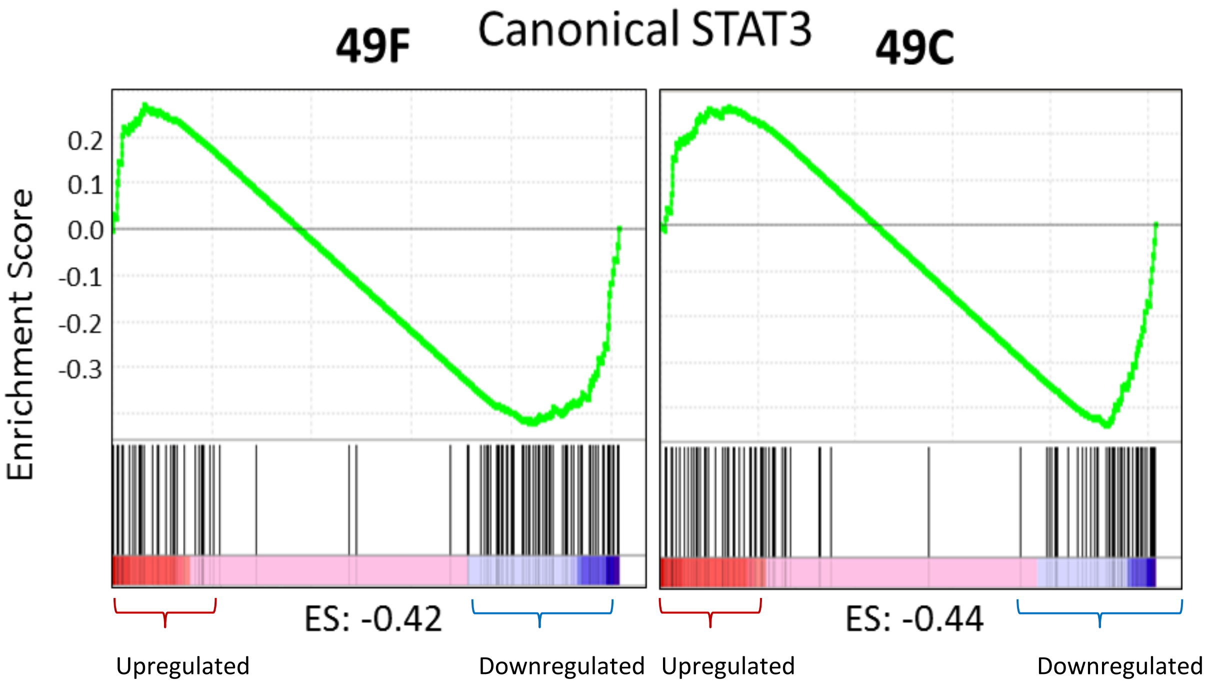

Supplementary Figure S3

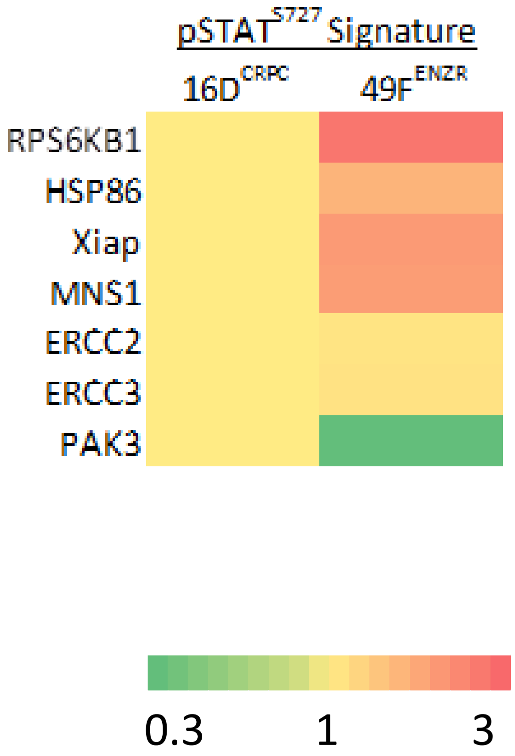

Supplementary Figure S4

A

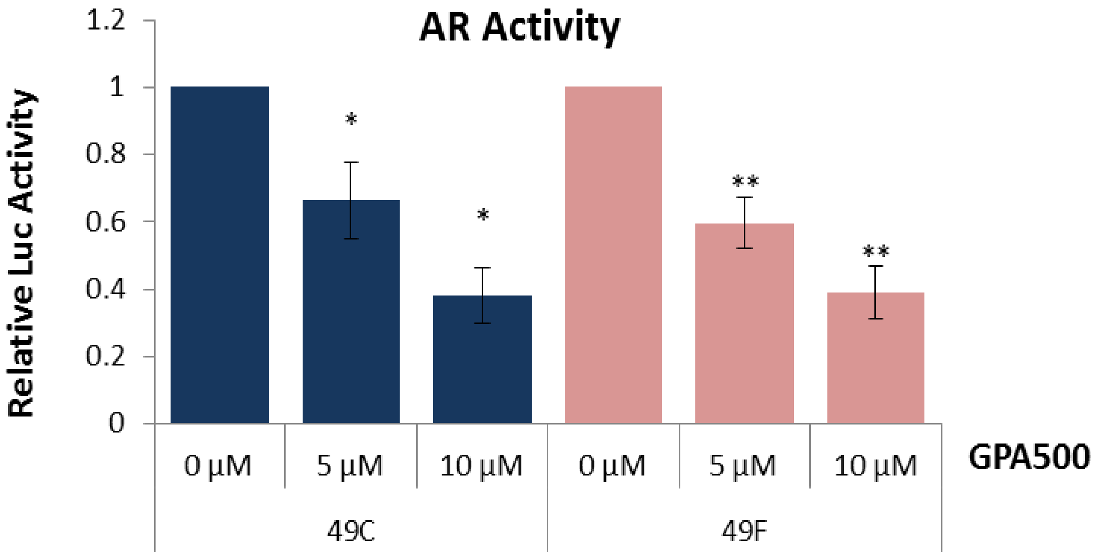

B

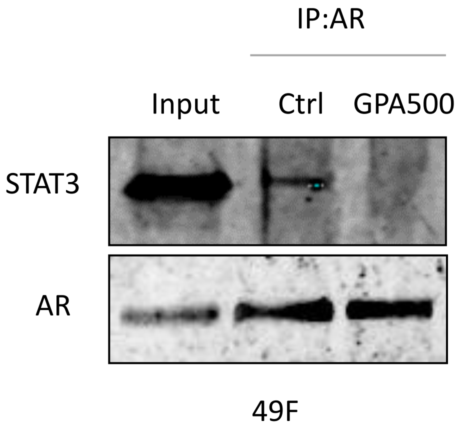

Supplementary Figure S5 (Full length blots for Figure 1)

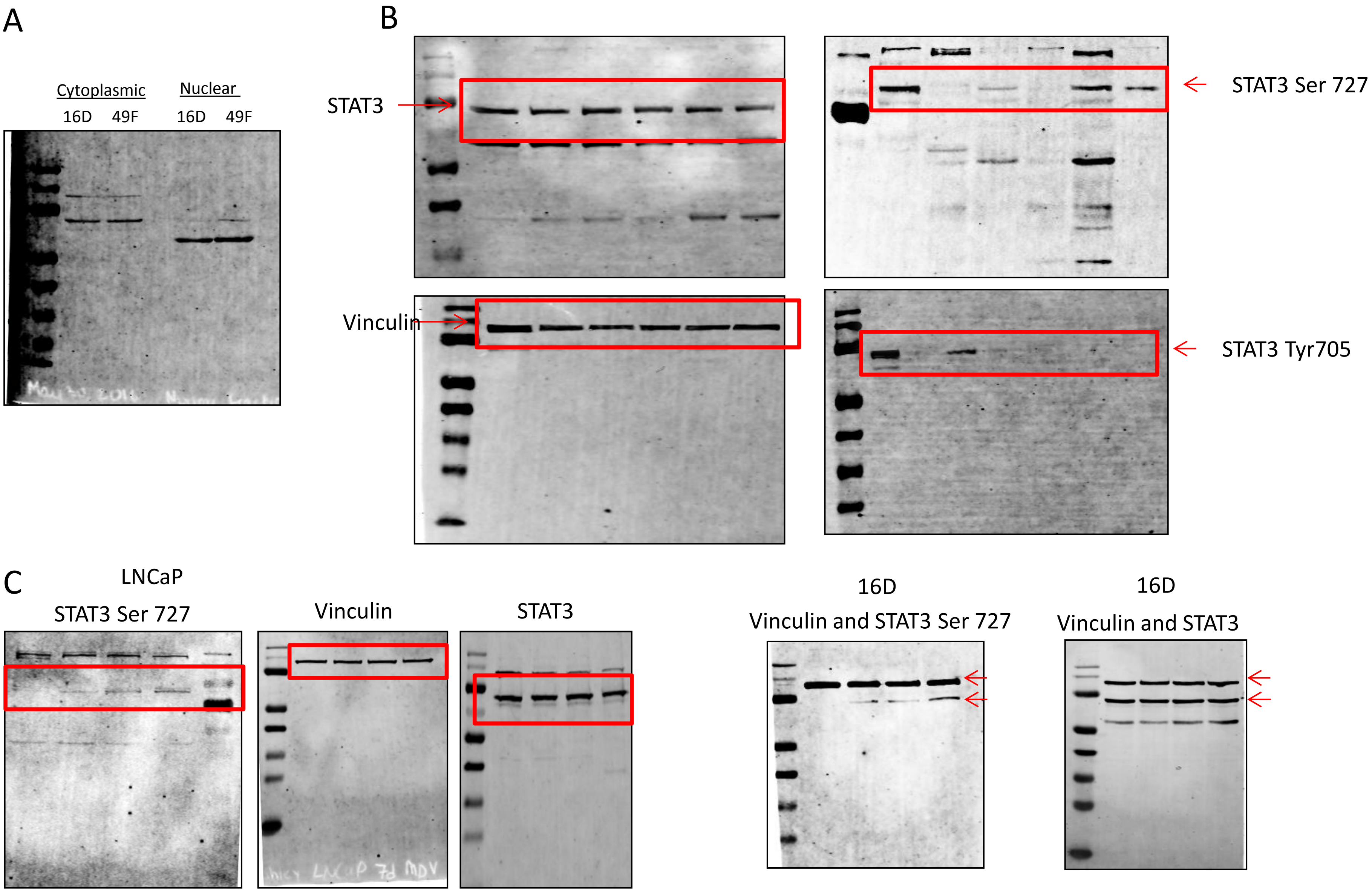

Supplementary Figure S6 (Full length blots for Figure 2)

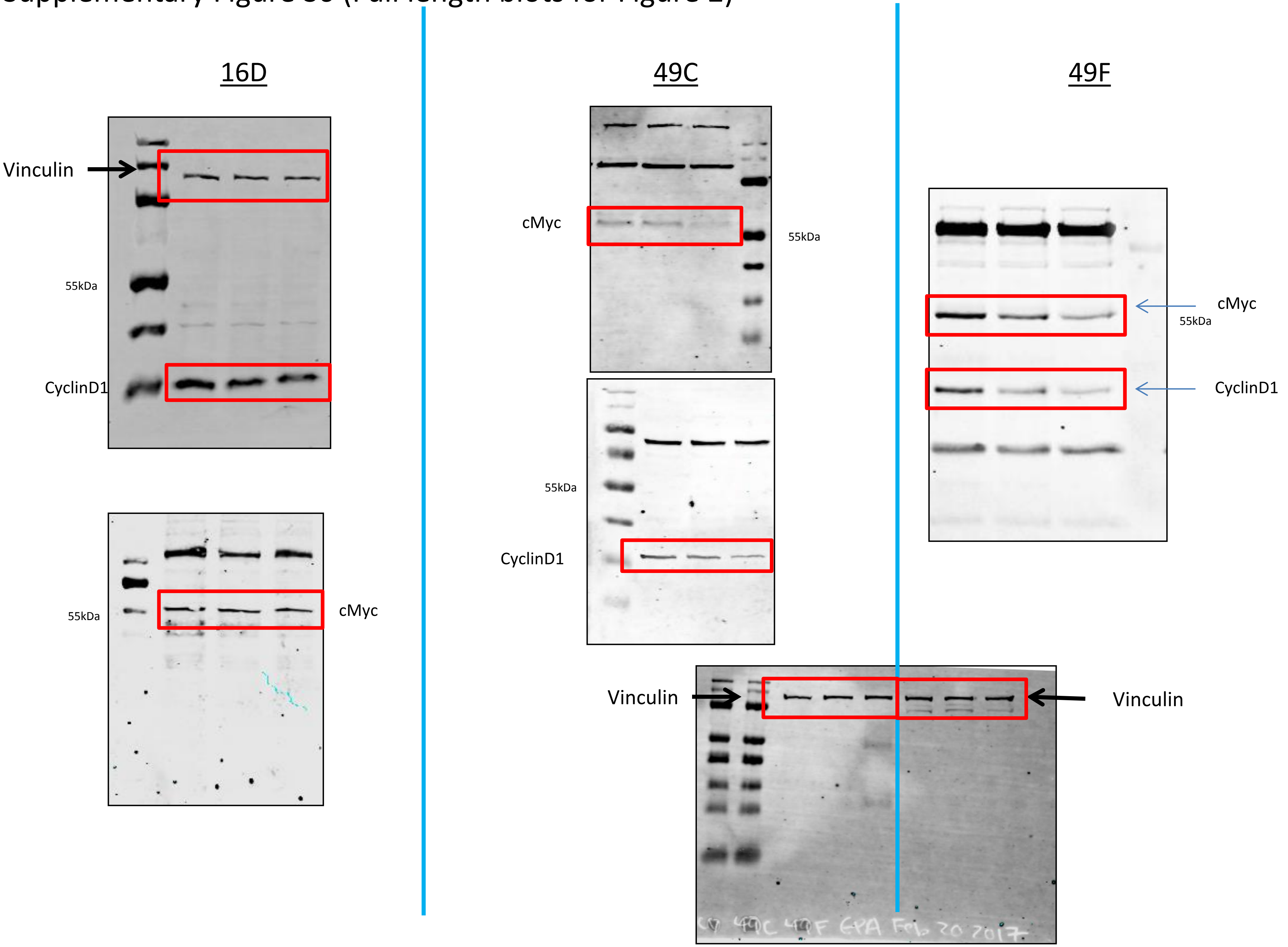

Supplementary Figure S7 (Full length blots for Figure 3b)

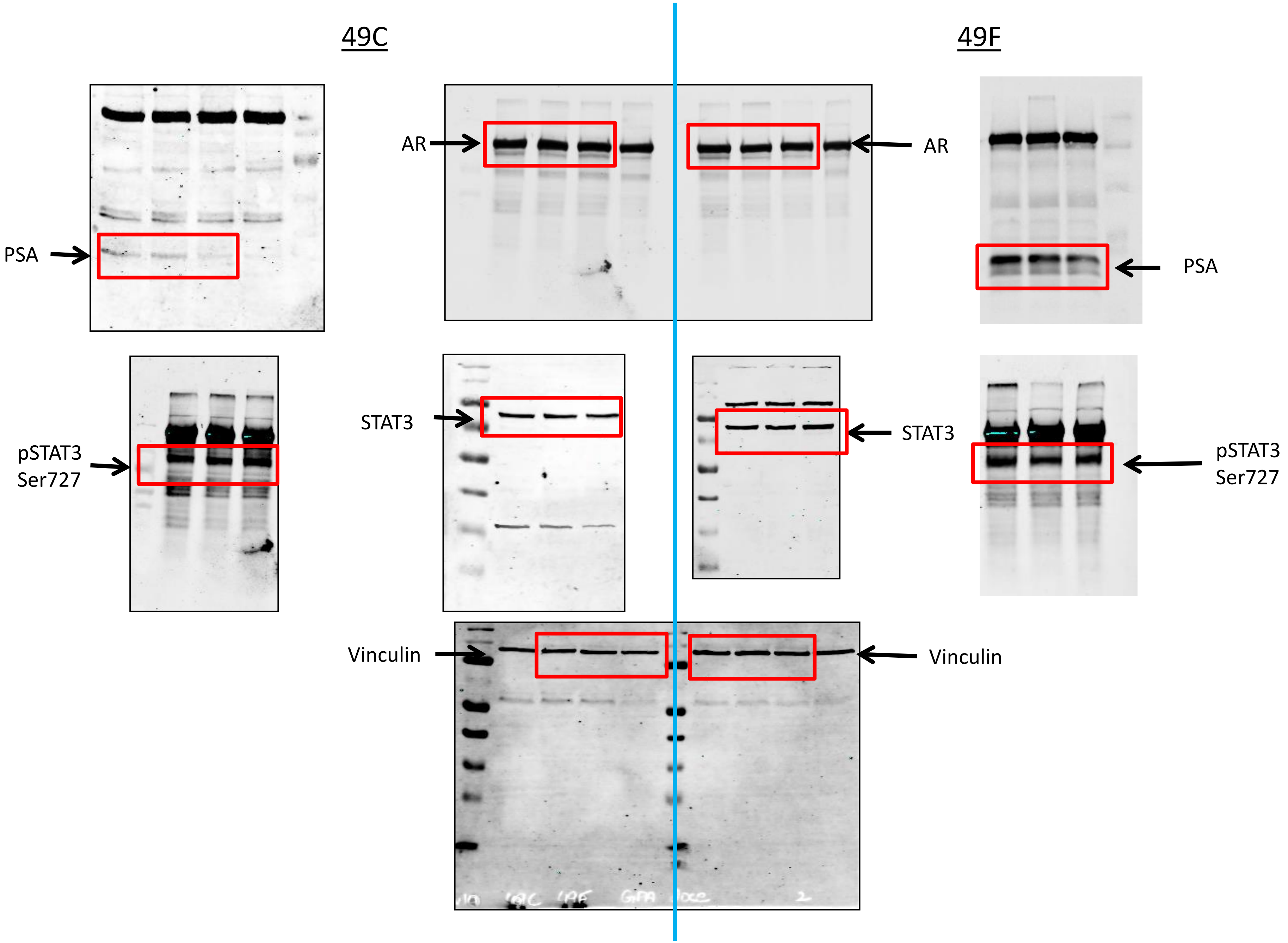

Supplementary Figure S8 (Full length blots for Figure 3c)

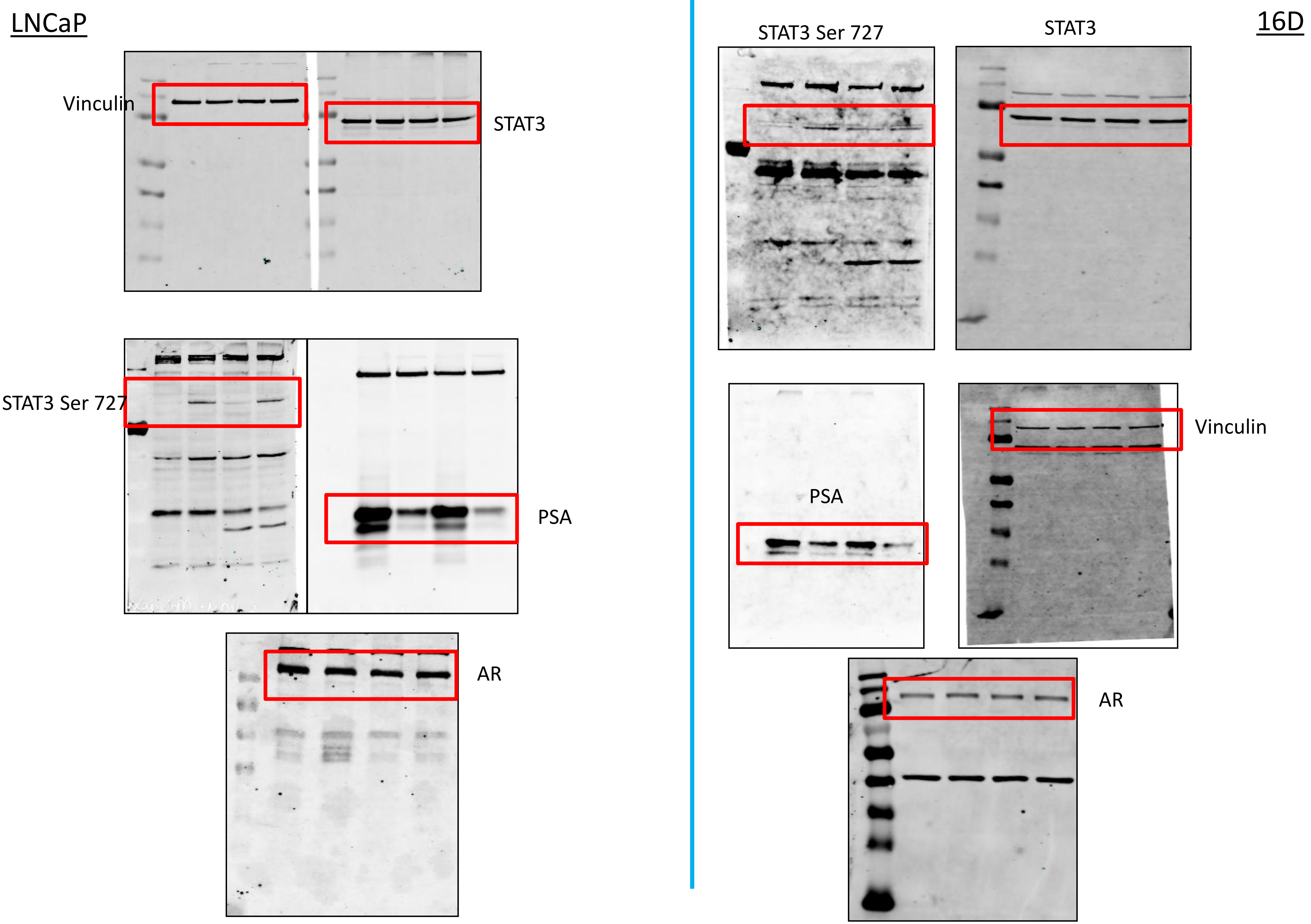

Supplementary Figure S9 (Full length blots for Figure 4)

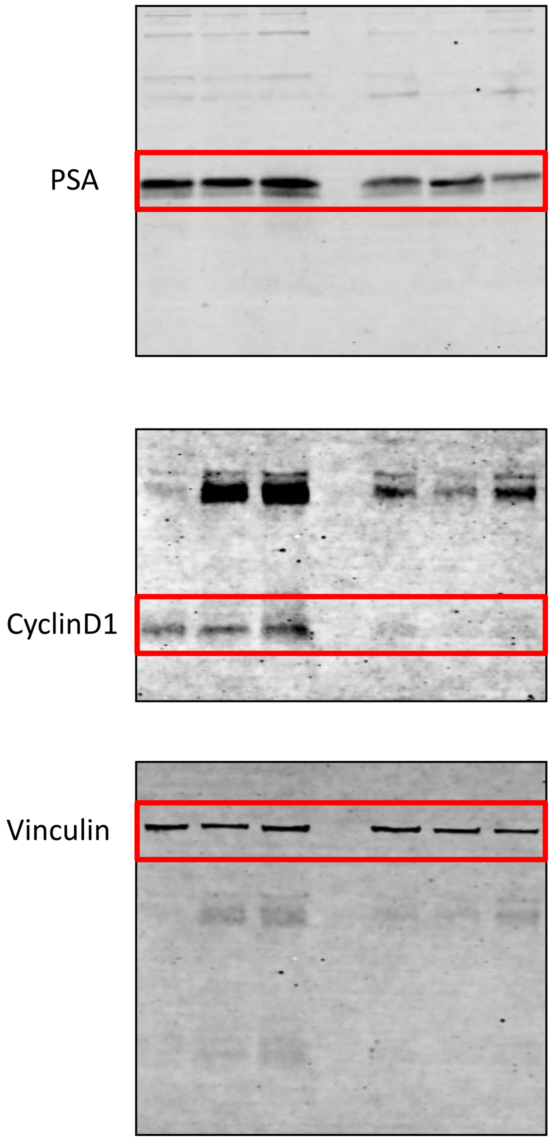

Mouse tumors

Supplementary Table 1: STAT3 canonical gene signature from Azare et. al (2007)

STAT3 up
AOX1
APOBEC3G
ARHGAP25
ARHGDIB
ARL4C
ARMCX2
B4GALT1
C3orf64
CA12
CALB1
CALD1
CALML3
CALU
CCND2
CDH11
CDH13
CDK17
CDV3
CHP
CLDN1
CLIC4
COL5A1
COL5A2
COPA
CPS1
CSF3
CUX2
CXCL11
CYP51A1
D4S234E
DCBLD2
DDX3X
DDX58
DHX9
DPYSL3
DUSP4
ELAVL2
EMP3
FEZ1
FGFBP1
FLI1
FN1

FOXA2
FST
G0S2
G3BP2
GABPB1
GNG11
GPX3
GREM1
HMGCS1
HRH1
IFI16
IFI27
IFI6
IFITM1
IL13RA1
IL13RA2
IPW
ITGB6
JAG1
KCNMA1
LPXN
LTBP1
MAN1A1
MAP1B
MCL1
MMP3
MX1
NEAT1
NEFL
NF2
NNMT
NUP160
NUP188
NUPR1
OAS1
OAS2
PALLD
PCDH7
PDPN
PID1
PLK2
PLTP
PRNP
PRSS3
PTGS2
PTPN11
PTPRK

RAB5A
RAB6A
ROBO3
ROD1
RPS4Y1
S100A8
S100A9
SCG5
SERPINE2
SFRP1
SH2D2A
SLC4A7
SORL1
SPOCK1
SRGN
SRSF6
SSR3
STX10
STX16
TGFA
TGFBR1
TGFBR2
THBS1
TNC
TP63
TWF1
VNN1
VSNL1
WDR1
WIPF1
XYLT1
YKT6

Supplementary Table 2:

STAT3 non-canonical gene expression in 49F ENZ resistant cells compared to 16D CRPC

Gene	16D^{CRPC}	49F^{ENZR}
RPS6KB1	1	1.900526
HSP86	1	1.430572
Xiap	1	1.625311
MNS1	1	1.606921
ERCC2	1	1.061115
ERCC3	1	1.073336
PAK3	1	0.32112

Supplementary Table 3: List of SYBR Green Primer Sequences for qRT-PCR

Name	Primer Sequences	
	Forward	Reverse
AR	TACCAGCTCACCAAGCTCCT	GCTTCACTGGGTGTGGAAA
bFGF	TGGTATGTGGCACTGAAACGA	TTCTGCCCAGGTCCTGTTTT
C-MYC	TGAGGAGACACCGCCCA	AACATCGATTTCTTCCTCA
FKBP51	TCCCTCGAATGCAACTCTCT	GCCACATCTCTGCAGTCAAA
GAPDH	CGACCTGACCTGCCGTCTAGAA	GGTGTCGCTGGTGAAGTCGAGAG
NKX3.1	GGACTGAGTGAGCCTTTTGC	CAGCCAGATTTCTCCTTTGC
PSA	CACAGCCTGTTTCATCCTGA	AGGTCCATGACCTTCACAGC
TMPRSS2	TCCCTCGAATGCAACTCTCT	GGATGTGTCTTGGGGAGCAA
VEGF	CCCACTGAGGAGTCCAACATC	GGCCTTGGTGAGGTTTGATC
